# Role for calcium‐activated potassium channels (BK) in migration control of human hepatocellular carcinoma cells

**DOI:** 10.1111/jcmm.16918

**Published:** 2021-09-12

**Authors:** Yuan He, Yingying Lin, Fei He, Lijuan Shao, Wei Ma, Fei He

**Affiliations:** ^1^ Department of General Surgery Changzhi Medical College Affiliated Heping Hospital Changzhi China; ^2^ Department of Immunology School of Basic Medical Sciences Fujian Medical University Fuzhou China; ^3^ Department of Stomatology The Second Clinical Medical College Shenzhen People’s Hospital Jinan University Shenzhen China; ^4^ Integrated Chinese and Western Medicine Postdoctoral Research Station Jinan University Guangzhou China; ^5^ Translational Medicine Collaborative Innovation Center of Shenzhen People’s Hospital The Second Clinical Medical College of Jinan University The First Affiliated Hospital of Southern University of Science and Technology Shenzhen China

**Keywords:** BK channels, hepatocellular carcinoma, migration, proliferation, xenografted mice

## Abstract

Hepatocellular carcinoma (HCC) is a leading cause of cancer‐related death worldwide. Its high metastasis rate is significantly correlated with poor patient prognosis. Elucidating the molecular mechanism underlying HCC metastasis is essential for HCC treatment. Owing to their high conductance, large‐conductance calcium‐activated potassium channels (BK channels) play a critical role in the control of membrane potential and have repeatedly been proposed as potential targets for cancer therapy. Emerging evidence suggests that BK channels are involved in the progression of cancer malignancies. The present study investigated the role of BK channels in mediating the hypoxia‐stimulated migration of HCC cells both in vitro and in vivo in the absence and presence of various BK channels modulators. We found that BK channels were functionally expressed on the membranes of the SMMC‐7721 and Huh7 HCC cell lines. Furthermore, blockage or activation of BK channels on the surface of HCC cells correspondingly inhibited or promoted HCC cell proliferation, migration and invasion in hypoxia conditions, with altered expression and distribution of cell‐cell adhesion molecule E‐cadherin and typical marker of mesenchymal cells, Vimentin, but not N‐cadherin. Hypoxia conditions did not alter BK channels expression but increased its open probability. Moreover, BK channels blocker IbTX significantly inhibited HCC cell remote colonization in HCC cell xenografted mice. In conclusion, the results of this study suggest that blocking BK channels offers an attractive strategy for treating HCC.

## 1 INTRODUCTION

Hepatocellular carcinoma (HCC) is highly prevalent worldwide; coupled with poor prognosis and it has become the second leading cause of cancer‐related death in China.^1^ Currently, the power of HCC diagnosis and treatment are limited because of its high degree of malignancy, rapid progression and metastasis to distance organs.^2^ Although numerous studies have assessed driver genes,^3^ signalling pathways and epithelial mesenchymal transition (EMT)^4^, ^5^ involved in cancer cell migration, how cancer cells move is still not fully understood. The present study provides a unique perspective to explain cancer cell mobility.

Ion channels on cell membranes are specialized proteins that facilitate the movement of specific ions across the plasma membrane. Ion channels are essential for basic cellular processes such as nerve impulses, cell proliferation, secretion of hormones and sensory transduction.^6^ However, they also play a role in in the abnormal progression of cancer, including limitless replicative potential, insensitivity to anti‐growth signals, tissue invasion and metastasis.^7^, ^8^ Among all the ion channels, potassium channels (K^+^ channels) represent the most diverse super‐families, with huge functional and structural diversity.^9^

Large‐conductance calcium‐activated potassium channels (BK channels, Maxi‐K channels, KCNMA1, KCa_1.1_, Slo1)^10^ belong to a voltage‐gated potassium channel family which contains other two member, intermediate‐conductance (IK)^11^ and small‐conductance (SK)^12^ calcium‐activated potassium channels. BK channels were first identified in chromaffin cells in 1981^13^ and were later found to be expressed in neurons across vibrate nerve systems.^14^, ^15^, ^16^ BK channels are activated by membrane depolarization and by elevated cytosolic Ca^2+^ levels. BK channels exist as a tetramer across the cell membrane and comprise four α‐subunits, either alone or coupled with β‐subunit pairs. The BK channels antagonist or blocker, iberiotoxin (IbTX), selectively binds to pore forming unit, α‐subunits,^10^ while BK channels agonist or opener NS1619 belongs to a synthetic benzimidazolone derivatives family that selectively activates the α‐subunit of BK channels. NS1619 was first found on specific activation of BK channels in smooth muscle cells.^17^

Recently, researchers have shown an increased interest in the role of ion channels in cancer development and progression. Firstly, BK channels are involved in cell cycle regulation and cell proliferation. Sizemore observed that opening large‐conductance potassium channels selectively induced cell death of triple‐negative breast cancer.^18^ Maqoud et al reported cell cycle regulation by Ca^2+^‐Activated K^+^ (BK) channels modulators in SH‐SY5Y neuroblastoma cells.^19^ Second, BK channels participated in cell migration and neoplastic of cancer cells. Recent evidence has shown that activation of BK channels contributes to PL‐induced mesenchymal stem cell migration.^20^ Moreover, the blockage of BK channels inhibits hypoxia‐induced migration and chemoresistance to cisplatin in human glioblastoma cells.^21^ Finally, overexpression of large‐conductance calcium‐activated potassium channels in human glioblastoma stem‐like cells and their role in cell migration has also been reported.^22^ Although it is now well established that BK channels are associated with cancer progression, their role in HCC has not been explored.

Our study aimed to explore the influence of BK channels on the malignant progression of HCC. To this end, we applied patch‐clamp techniques to identify BK channels in SMMC‐7721 and Huh7 cells. We investigated the effect of BK channels opener and blockers on cancer cell proliferation, migration and invasion under both normoxic and hypoxic conditions. We have demonstrated that BK channel blocker played an important role in the inhibition of HCC growth and metastasis in vivo, suggesting that these channels may represent a promising candidate therapeutic target for HCC.

## 2 MATERIALS AND METHODS

### 2.1 Cell culture and reagents

The HCC cell lines SMMC‐7721 and Huh7 were purchased from Cell Bank of Shanghai, Institutes for Biological Sciences, China. Huh7 cells and LO2 cells were cultured in Dulbecco's Modified Eagle's Medium (Hyclone, #SH30243) supplemented with 10% foetal bovine serum (Gibco, # 10099–141), and SMMC‐7721 cells were feed in RPMI 1640 Medium (Hyclone, # SH30809.01B) with 10% foetal bovine serum in a humidified incubator at 37℃ and 5% CO_2_. Cells were disassociated by 0.25% trypsin/EDTA (Invitrogen, #25300‐120) and passed at a density of 1:4–1:5 every three days. For patch‐clamp recording experiments and immunofluorescence assays, cells were seed on glass coverslips.

### 2.2 Electrophysiology

Whole‐cell currents were recorded using an EPC‐10 amplifier (HEKA Elektronic, German) following standard recording techniques as described previously.^15^, ^16^ The pClamp 10.0 software (Axon Instruments. USA) was employed to acquire and store data. Patch pipettes were made of thin‐walled borosilicate glass (model P‐10, Narishige) using a P‐97 horizonal micropipette puller (Sutter Instruments, USA). Pipette resistances were 2–5 M Ohm filled with a solution composed of (in mM) 130 KCl, 1 CaCl_2_, 2 MgCl_2_, 10 EGTA, 10 HEPES and pH 7.4. The bath solution for whole‐cell recordings contained (in mM) 150 NaCl, 5 KCl, 2 CaCl_2_, 1 MgCl_2_, 10 HEPES and pH 7.4. All the experiments were done in room temperature (20–22℃). The value of the single‐channel open probability (P_0_) in a patch with multiple channels was calculated by using TAC 4.1 (HEKA, Germany), based on the equation: P_0_ = (1 − Pc^1/N^), where Pc is the probability when all of the channels are in the closed state, N is the number of channels in the patch.

### 2.3 Cell proliferation assay

Cell proliferation was detected using a Cell Counting Kit‐8 (Solarbio, #YZ‐CK04‐500T), according to the manufacturer's instructions. The CCK‐8 assay was conducted to check the effect of BK channel blockers and opener on cell growth in medium without serum under both normoxic and hypoxic conditions.

### 2.4 Wound‐healing assay

HCC cells were plated in 6‐well plates coated with collagen in DMEM with or without 10% serum. When the cells reached nearly 90% confluence, a linear wound was made by straightly scratching the cell monolayer with a sterilized 10μL pipette tip. Then, cells were cultured with fresh medium for another 48 h in the presence of BK channel modulators (NS1619 10 μM, IBTX 10 nM and TEA 10 mM). Cells treated with DMSO were used as controls. The area of the wound gap (blank area) was then observed and photographed under an inverted microscope (20×) (Leica, #DMi8, Germany), and the area of the wound gap was measured by image J software to indicate cell migration ability. All studies were performed with three replicates.

### 2.5 Cell migration and invasion assays

Migration of HCC cells was assessed using the 24‐well polycarbonate membrane cell migration assay kit (Corning Incorporated Costar, #3422, USA). The invasion assay was performed in a similar fashion using BD BioCoat™ Matrigel™ Invasion Chambers (BD Biosciences, #354480) as previously described.^23^ The transwell assays were conducted in 10 µg/ml mitomycin C (Sigma‐Aldrich, #M5353) containing medium which often used as a chemotherapeutic agent by virtue of its anti‐proliferation activity. Each experiment was performed in triplicate.

### 2.6 Imaging assays

HCC cells for immunofluorescence assays were grown in 6‐well plates with treatment of BK blockers. Then, cells were fixed for 20 min in 4% (w/v) paraformaldehyde at room temperature, followed by 3 times PBS washing. After that, fixed cells were treated with 0.1% (v/v) Triton X‐100 (Sigma‐Aldrich, #T9284) for 15 min for permeation and then blocked for 60 min with 5% (v/v) normal serum in PBS. Permeabilized cells were incubated with primary antibodies at 4℃ overnight and secondary antibodies for 1 h at room temperature, followed by washing and staining with 1 μg/ml DAPI (Sigma‐Aldrich, #D8417). Alexa Fluro secondary antibodies from Molecular Probes were used at 1:1000. Images were taken using a Leica invert microscope (Leica, #DMi8) with its software LAS X.

### 2.7 Western blot

Western blot was performed as previously described.^24^ Total protein was extracted from cells in RIPA lysis buffer (Beyotime, #P0013B) and quantified using a Bradford assay. In total, 30 μg of protein was separated using 10% sodium dodecyl sulphate‐polyacrylamide gel electrophoresis (SDS‐PAGE) and then transferred to a polyvinylidene difluoride (PVDF) membrane (Millipore, #ISEQ0010). The membrane was blocked in a 5% powdered milk solution and incubated in primary antibody overnight at 4℃. After washing, the membrane was incubated with a horseradish peroxidase‐conjugated secondary antibody (dilution 1:4000) at 37℃ for 1 h. Protein bands were visualized using Western Bright ECL (Millipore, #WBKLS0500) and detected using ImageQuant LAS4000‐mini (General Electric, USA). Relative protein levels were calculated based on a β‐Actin loading control. The antibodies used for Western blot were listed below: anti‐BK channel antibody (Alomone Labs, APC‐151, dilution 1:500), anti‐HIF1α antibody (Cell Signaling Technology, #36169, dilution 1:1000), anti‐N‐cadherin antibody (Cell Signaling Technology, #13116, dilution 1:1000), anti‐E‐cadherin antibody (Cell Signaling Technology, #3195, dilution 1:1000), anti‐Vimentin antibody (Cell Signaling Technology, #5741, dilution 1:1000) and Anti‐β‐Actin Mouse Monoclonal Antibody (TransGen Biotec, #HC201‐01, 1:4000).

### 2.8 Quantitative real‐time reverse transcription polymerase chain reaction

Total RNA was extracted from cells by TRIzol^®^ Reagent (Life Technologies, #15596‐018) according to manufacturers’ instructions. Then, the complementary DNA was generated and qPCR analysis was done as previously described.^23^ The PCR primers are listed in Supplementary file 1: Table S1.

### 2.9 Synchronization of the cells and cell cycle analysis.

HCC cells were synchronized in the G0 phase of the cell cycle by serum starvation for 24h. The stimulation of the cell proliferation was performed by serum addition. After initiation of cell proliferation, cell cycle phase distribution was analysed at different time points. The adherent HCC cells were rinsed with PBS, harvested using trypsin‐EDTA solution and suspended in the growth medium. Cells were permeabilized with 0.1% Triton X‐100 (Sigma, #T8787) and stained for 5 min with 2 μg/ml DAPI. Cell cycle phase distribution was measured with CytoFLEX flow cytometer (Beckman Coulter, USA) with at least 20,000 events recorded for each test. Analysis of the results was performed using FlowJo VX software.

### 2.10 Hypoxic conditions and drugs

Hypoxic conditions for cell culture were achieved by a 2.5‐litre Pack‐Rectangular Jar (#C‐31), AnaeroPack (#C‐1) and Oxygen indicator (#C‐22) purchased from MITSUBISHI GAS CHEMICAL COMPANY, INC. BK channel agonist NS1619, (#3804, TOCRIS), and antagonist Iberiotoxin (Sigma‐Aldrich, #5904, IbTX) were dissolved in dimethyl sulphoxide (Sigma‐Aldrich, # D8418, DMSO), and the working solutions were prepared by adding a necessary amount of stock to the culture media.

### 2.11 Analysis the function of BK channel blocker and opener in HCC tumour xenografted mice

All animal protocols were approved by the Animal Care and Use Committee of Shenzhen People's Hospital. Male BALB/c nude mice (4 weeks old) were purchased from Guangzhou Experimental Animal Center of Chinese Academic of Sciences (Guangzhou, China). We kept the mice under standard pathogen‐free conditions. The mice were allowed to acclimate for 7 days before use. SMMC‐7721 cells (2 × 10^6^/0.15 ml of PBS) were subcutaneously injected into right flank of each mouse (n=6 mice/group). 4 weeks later, the drugs were injected into the tail veins of the mice till the mice were sacrificed. Tumour growth was monitored once a week using a caliper, and the tumour volume was calculated using the following formula: volume =π/6 × length×width^2^.

### 2.12 Statistical analyses

All statistical analyses were performed using GraphPad Prism 6 software. A *p*‐value<0.05 was considered statistically significant.

## 3 RESULTS

### 3.1 BK channels are functionally expressed in hepatocellular carcinoma cell lines

**FIGURE 1:**
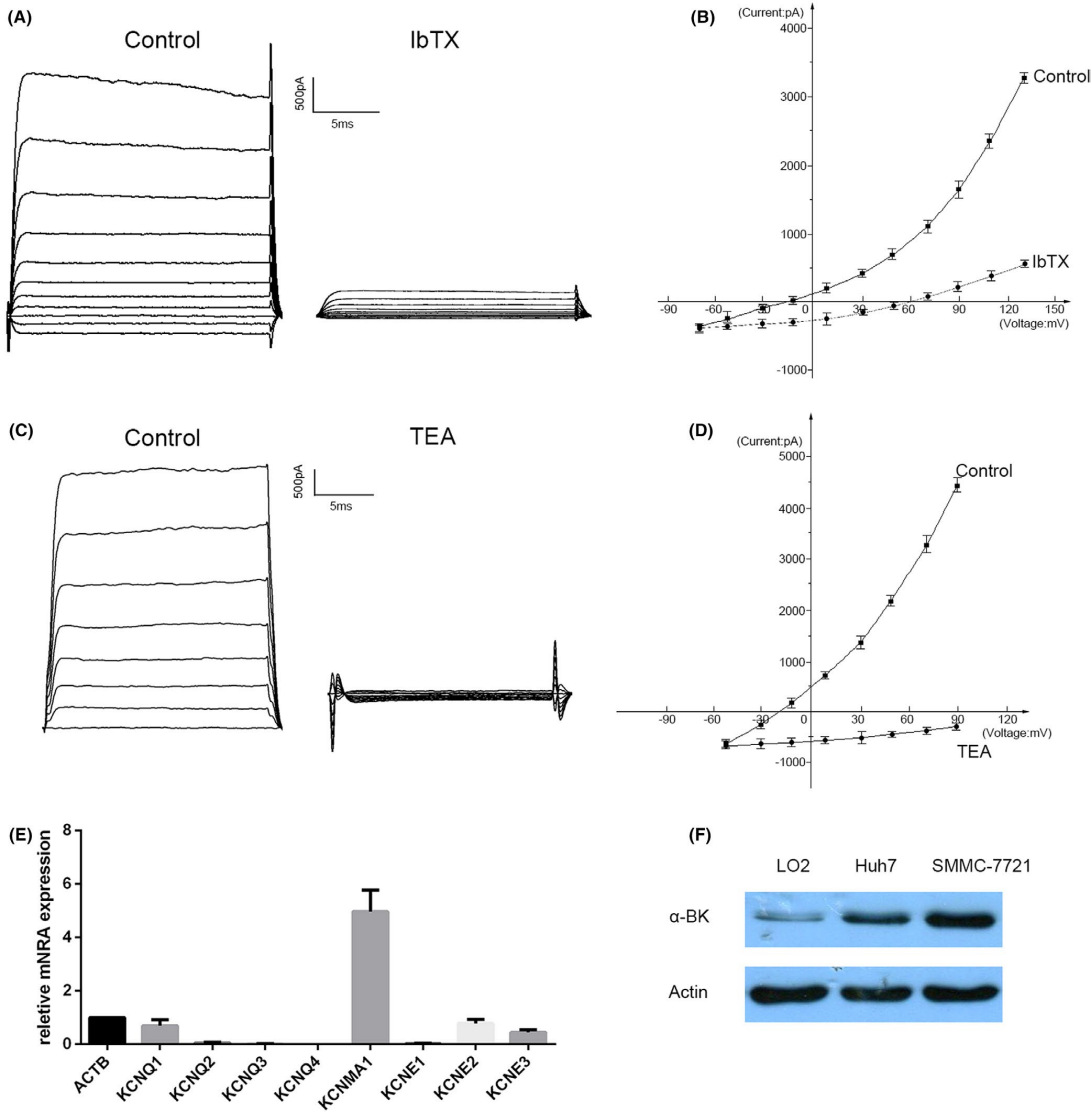
BK channels were functionally expressed in HCC cells. (A). Representative whole‐cell currents of SMMC‐7721 cells evoked after voltage steps from –70 mV to +130 mV for 20 milliseconds with 20 mV increment. (B). Whole‐cell current‐voltage relationship in SMMC‐7721 cells in the absence and presence of 100 nM IbTX. (C). Representative whole‐cell currents of SMMC‐7721 cells evoked after voltage steps from –70 mV to +90 mV for 15 milliseconds with 20 mV increment. (d). whole‐cell K^+^ currents were potently suppressed by 10 mM TEA. (E) Real‐time PCR analysis of BK channels and other potassium channels in SMMC‐7721 cells. (F) Western blot analysis of α subunit of BK channels in LO2, Huh7 and SMMC‐7721 cells

Alterations of ion channels on the surface of cell membranes may affect the progression malignant cancer.^8^, ^9^ The present study aimed to investigate the role of BK channels in HCC progression. We examined the expression of BK channels in two typical HCC cell lines, Huh7 and SMMC‐7721. First,we recorded whole‐cell currents from SMMC‐7721 cells using the configuration of the patch clamp. These whole‐cell currents were mainly BK channel currents as they were largely blocked by IbTX (Figure 1A,B). Besides, the whole‐cell currents were completely diminished with tetraethylammonium (TEA) treatment, which non‐selectively blocks K^+^ currents (Figure 1C,D). Likewise, we obtained similar results of whole‐cell current in Huh7 cells (Figure S1). Consistent with the electrophysiological data, quantitative polymerase chain reaction (qPCR) analysis revealed high expression of the KCNMA1 transcript coding for the BK channels (Figure 1E), while other potassium channels such as KCNQ1, KCNE2 and KCNE3 were poorly expressed in HCC cells. Moreover, the α‐subunit of BK channels was detected in LO2, Huh7 and SMMC‐7721 cells by Western blot (Figure 1F). In addition, the whole‐cell current recorded in LO2 cells was typical BK channel current as it could be blocked by IbTX (Figure S2). These results indicated that BK channels are functionally expressed on HCC cells and normal liver cells and suggest they play a significant role on control of the HCC cell membrane currents.

### 3.2 The inhibitory effect of the BK channel blockers on HCC cell proliferation and clone formation

**FIGURE 2:**
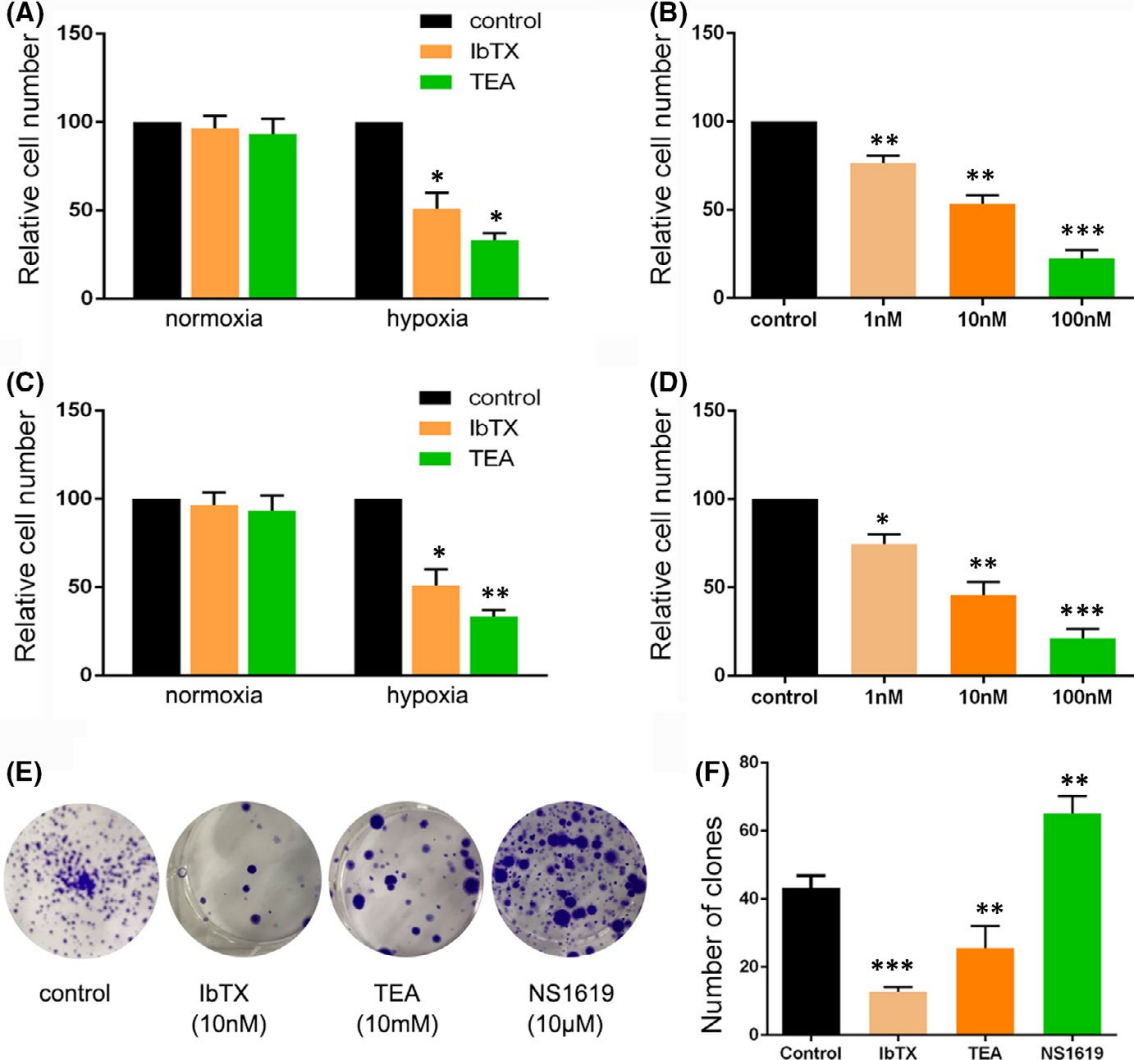
Effects of BK channel inhibition on proliferation of HCC cells under normoxic and hypoxic conditions. (A) Relative cell numbers of SMMC‐7721 cells assayed by Cell Counting Kit‐8 in control group, IbTX group and TEA group. (B) Relative cell numbers of SMMC‐7721 cells with the treatment of different concentration of IbTX under hypoxic culture condition. (C) Relative cell numbers of Hun7 cells assayed by Cell Counting Kit‐8 in the control, IbTX and TEA groups. (D) Relative cell numbers of Huh7 cells with the treatment of IbTX in different concentration under hypoxic culture condition. (e‐f) Colony formation assay of SMMC‐7721 cells in control, IbTX, TEA and NS1619 groups. Each experiment was done in triple replicate, and *p*<0.05, *p *< 0.01 and *p *< 0.001 were marked *, ** and*** respectively

To determine whether BK channels affect the HCC cell proliferation, we evaluated cell viability using CCK‐8 assay coupled with the IbTX, TEA and NS1619 treatment for 72 h. In the SMMC‐7721 cells, neither 10 mM TEA nor 10nM IbTX changed cell viability under normoxic conditions; however, both TEA and IbTX reduced cell viability under hypoxic conditions (Figure 2A), with IbTX reducing cell viability in a dose dependent manner (Figure 2B). Likewise, the BK channels blocker IbTX and TEA did not significantly alter cell viability of Huh7 cells significantly under normoxic conditions but decreased cell proliferation ability in hypoxia conditions (Figure 2C,D). In contrast, the BK channels opener NS1619 (10μM) drastically enhanced cell proliferation ability under hypoxic conditions (Figure S3.a) but did not affect HCC cell viability under normoxic conditions. Likewise, NS1619 merely upregulates normal liver cell (LO2 cell) proliferation under hypoxic conditions (Figure S3b). Thus, BK channel modulators showed different patterns of regulating HCC cell proliferation.

### 3.3 Blockage of BK channel induced G2 phase arrest in HCC cells

**FIGURE 3:**
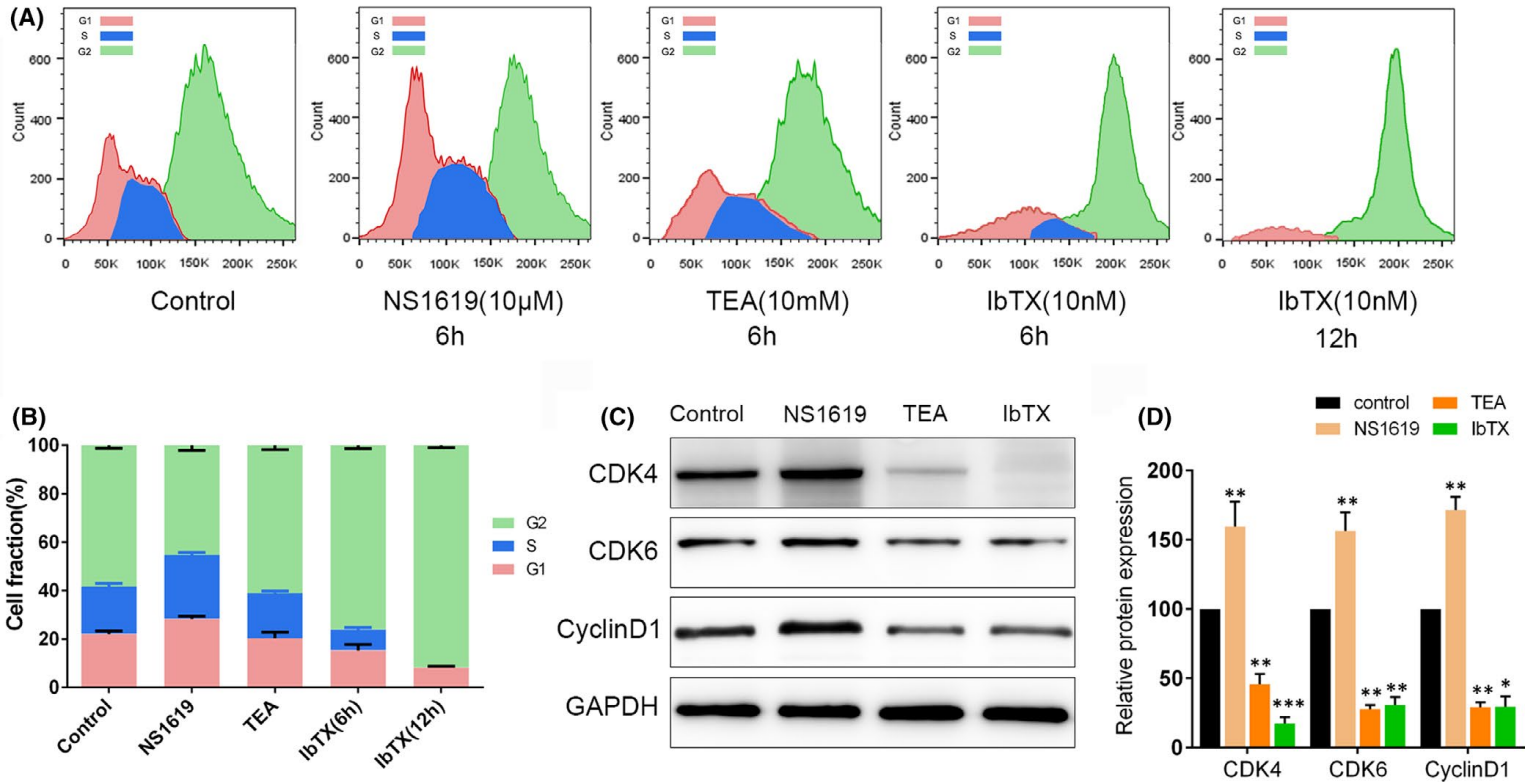
Blockage of BK channel induced G2 phase arrest of SMMC‐7721 cells. (A) Cell cycle was determined by flow cytometry for different experimental groups. No treatment (control), BK channel opener NS1619 treatment for 6 h, non‐selective blocker of K^+^ channels TEA treatment for 6 h, Selective blocker of BK channels IbTX treatment for 6 h and 12 h. (B) Cell percentages for different phases in the six experimental groups were shown in the diagram. Pink: G1 phase; Blue: S phase; Green: G2 phase. (C, D) The expression of G1 phase related proteins CDK4/6 and cyclinD1 was detected by Western blot, and *p*<0.05, *p*<0.01 and *p*<0.001 were marked *, ** and*** respectively

While switching on or off BK channel affected on HCC cell proliferation, the involvement of BK channels in cell cycle control of HCC cells required further investigation. We used a cell cycle assay to investigate the effect of BK channel blockers and opener on cell cycle progression. HCC cells were synchronized in the G0 phase by serum starvation before cell cycle assay. Then, the cells were then treated with the compounds at different time points and harvested for the assay. IbTX, TEA and NS1619 induced SMMC‐7721 cell cycle progression alterations and with different patterns. In detail, the BK channel opener NS1619 caused a reduction in G2 phase cell population, from 58.3% to 45.3%, along with a slight increase of G1 phase and S phase cells (Figure 3A,B). On the contrary, the non‐selective BK channel blocker TEA induced a G2 phase accumulation from 45.3% to 61.1% and was also associated with a decrease distribution in G1 phase (Figure 3A,B). Similarly, the treatment of selective external membrane impermanent BK channel blocker IbTX 6h and 12h produced an enlargement of G2 cell proportion from 58.3% to 76.1% and 91.9%, respectively, along with a large contraction of G1 and S cells (Figure 3A,B). The expression of cyclin D1, CDK4 and CDK6 was detected by Western blot. These three proteins mainly expressed in G1 phase.^25^ The results demonstrated that BK channel blockers TEA and IbTX reduced expression of cyclin D1 and CDK4/6. In contrast, NS1619 increased cyclin D1 and CDK4/6 expression, suggesting that NS1619 caused G2 arrest coupled with G1 phase shrinkage (Figure 3C,D).

### 3.4 Blocking BK channel inhibits HCC cell migration and invasion

**FIGURE 4:**
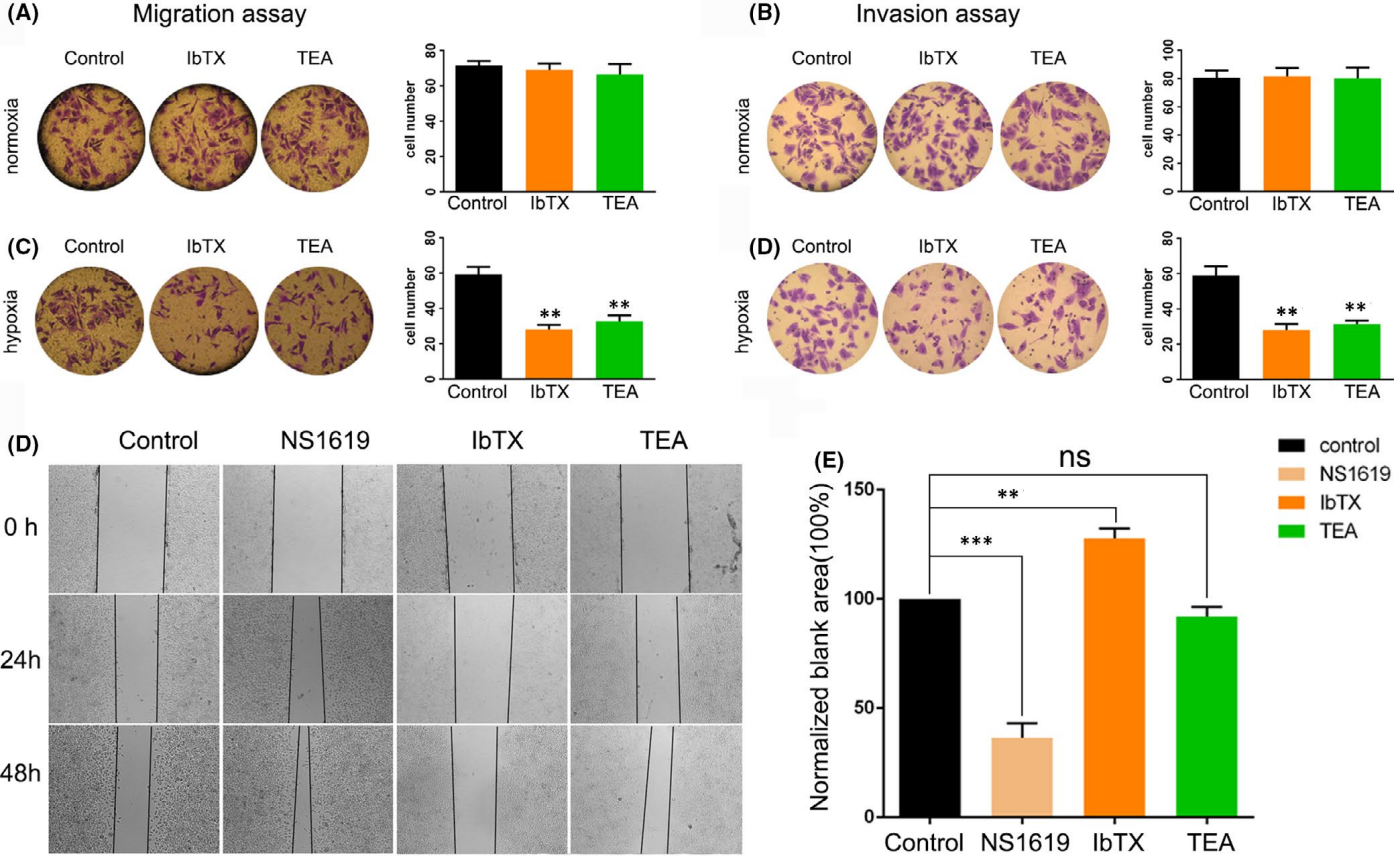
Effects of BK channel inhibition on migration of SMMC‐7721 cells under normoxic and hypoxic conditions. (A, C) Representative pictures of SMMC‐7721 cell migration in control, IbTX and TEA groups, in normoxic and hypoxic conditions (left panel), and quantitative analysis of migration assay (right panel). (B, D) Representative pictures of SMMC‐7721 cell invasion in control group, IbTX group and TEA group in normoxic and hypoxic conditions (left panel), and quantitative analysis of invasion assay (right panel). (E) Representative pictures of SMMC‐7721 cell in wound‐healing assay at 0, 24 and 48 h. (F) Quantitative analysis of wound‐healing assay. Each experiment was done in triple replicate, and *p*<0.05, *p*<0.01 and *p*<0.001 were marked *, ** and*** respectively

To investigate the function of BK channel in HCC metastasis, we employed transwell assay and wound‐healing assay with the treatment of BK channel opener and blockers. SMMC‐7721 cell migration and invasion were not affected by BK channel blockage mediated by IbTX and TEA under normoxic conditions (control=71, IbTX=69 and TEA=66; control=80, IbTX=81 and IbTX=80; Figure 4.a, b). However, IbTX and TEA treatment significantly reduced SMMC‐7721 cell migration and invasion (control=59, IbTX=28 and TEA=32; control=59, IbTX=28, TEA=31; Figure 4C, D) under hypoxic conditions. Likewise, BK channel opener NS1619 did not affect cell migration (control=118, NS1619=122, Figure S4) under normoxic condition but drastically increased SMMC‐7721cell migration (control=118, NS1619=244, Figure S4). Moreover, the results of wound‐healing assay confirmed the effect of BK channel opener and blockers on HCC cell migration. NS1619 significantly increased, and IbTX largely reduced the SMMC‐7721 cell migration as the relative blank area of the two groups differed statistically from that of the control group (NS1619=36.5 vs IbTX=127.7 vs control=100, Figure 4. e, f). Nevertheless, TEA did not significantly affect SMMC‐7721 cell migration as the relative blank areas of control group and TEA group were quite close (TEA=92.1 vs control=100.2, Figure 4E,F).

### 3.5 Hypoxia conditions do not alter BK channel expression but increased its open probability

**FIGURE 5:**
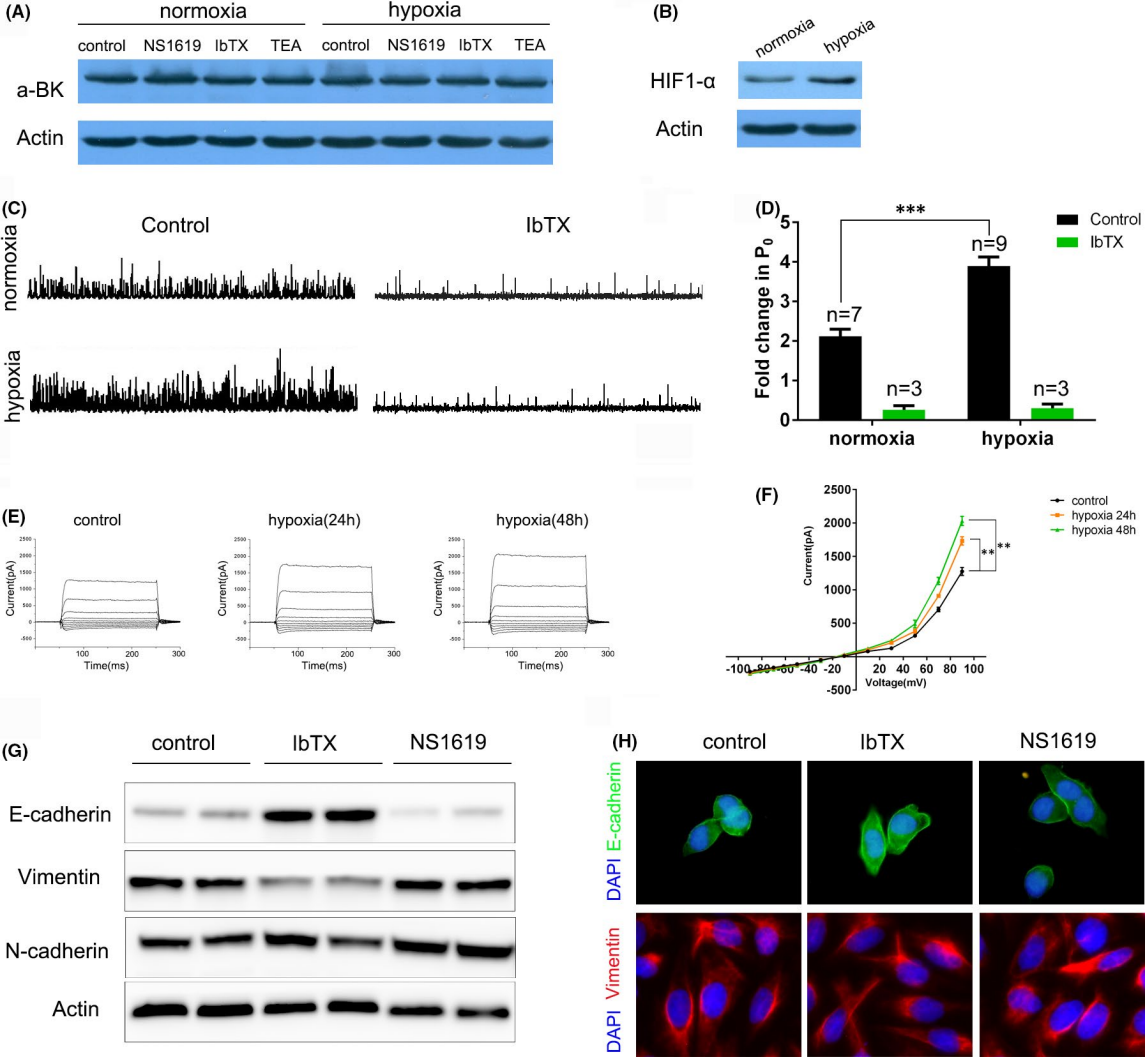
BK channel expression and function under normoxic and hypoxic conditions. (A) α‐BK and actin protein expression in SMMC‐7721 cells was analysed by Western blot under both normoxic and hypoxic conditions. (B) HIF1‐α and actin expression was examined by Western blot. (C) Representative single‐channel current trace of BK channel in normoxic and hypoxic groups, and (D) statistical comparison of the fold change in P_0_ between normoxic and hypoxic cells. (E) Representative whole‐cell currents evoked after voltage steps from –90mV to +90 mV for 200 milliseconds with 20 mV increment for the control, hypoxia 24h and hypoxia 48h groups respectively. (F) Current‐voltage curve for e panel. (G) Western blot analysis of EMT related proteins in SMMC‐7721 cells in control, IbTX and NS1619 groups respectively. (H) Immunofluorescence analysis of the protein expression of E‐Cadherin and Vimentin with the treatment of DMSO, IbTX and NS1619 respectively. Each experiment was done in triple replicate, and *p *< 0.01 and *p *< 0.001 were marked ** and*** respectively. P_0_ refers to single‐channel open probability of BK channel

Our findings that BK channel blockers and opener regulate cell proliferation and migration only under hypoxia conditions led us to speculate that hypoxia may play an important role mediating BK channel and HCC malignance. First, we examined the protein level of BK channel in SMMC‐7721 cells under both normoxic and hypoxic conditions. The result of western blot showed no difference of BK protein level among control, NS1619, IbTX and TEA group, either under normoxic nor hypoxic conditions (Figure 5A). In addition, the expression of hypoxia‐induced factor 1‐α (HIF1‐α) was increased under hypoxic conditions (Figure 5B), which indicated the successful establishment of hypoxic conditions in our study. We then examined the effect of normoxic and hypoxic conditions on the single‐channel properties of BK channel in inside‐out patches. Representative single BK channel traces at the voltage of 40 mV in normoxia and hypoxia cells were illustrated in Figure 5C, with single‐channel open probability(P_0_) of BK channel of 2.1 and 3.8 in control groups respectively. The mean fold changes in P_0_ were 2.17 ± 0.05 for normoxic cells (n = 7) and 3.77 ± 0.09 for hypoxic cells (n = 9) respectively (Figure 5D). The whole‐cell patch recordings were performed to determine whether hypoxia affects the whole‐cell current of HCC cells. SMMC‐7721 cells with hypoxia treatment for 24h and 48h showed significantly increased whole‐cell currents, which are typical BK currents (Figure 5E). At a voltage of +90mV, the current was increased by hypoxia treatment from 1275 ± 33.86 pA to1732 ± 36.25 pA, 2031 ± 39.55 pA, for hypoxia lasting 24h and 48h respectively (Figure 5F). Together, these results indicated that the hypoxia treatment increased the single‐channel open probability and whole‐cell current of BK channel in HCC cells. To further investigate the potential mechanism underlying these findings, we analysed the EMT related proteins E‐cadherin, Vimentin and N‐cadherin by western blot. We found that IbTX induced higher expression of the epithelial protein E‐cadherin, while NS1619 reduced E‐cadherin expression compared to the control group. However, Vimentin decreased in IbTX treated cells compared to those in control groups and NS1619 groups. N‐cadherin expression was comparable in all the three groups (Figure 5G). In addition, immunostaining assay showed much higher E‐cadherin expression in IbTX treated cells compared to other two groups. Moreover, Vimentin was slightly decreased in IbTX group (Figure 5H).

### 3.6 Blockage of BK channel regulates growth of HCC cell xenograft in mice

**FIGURE 6:**
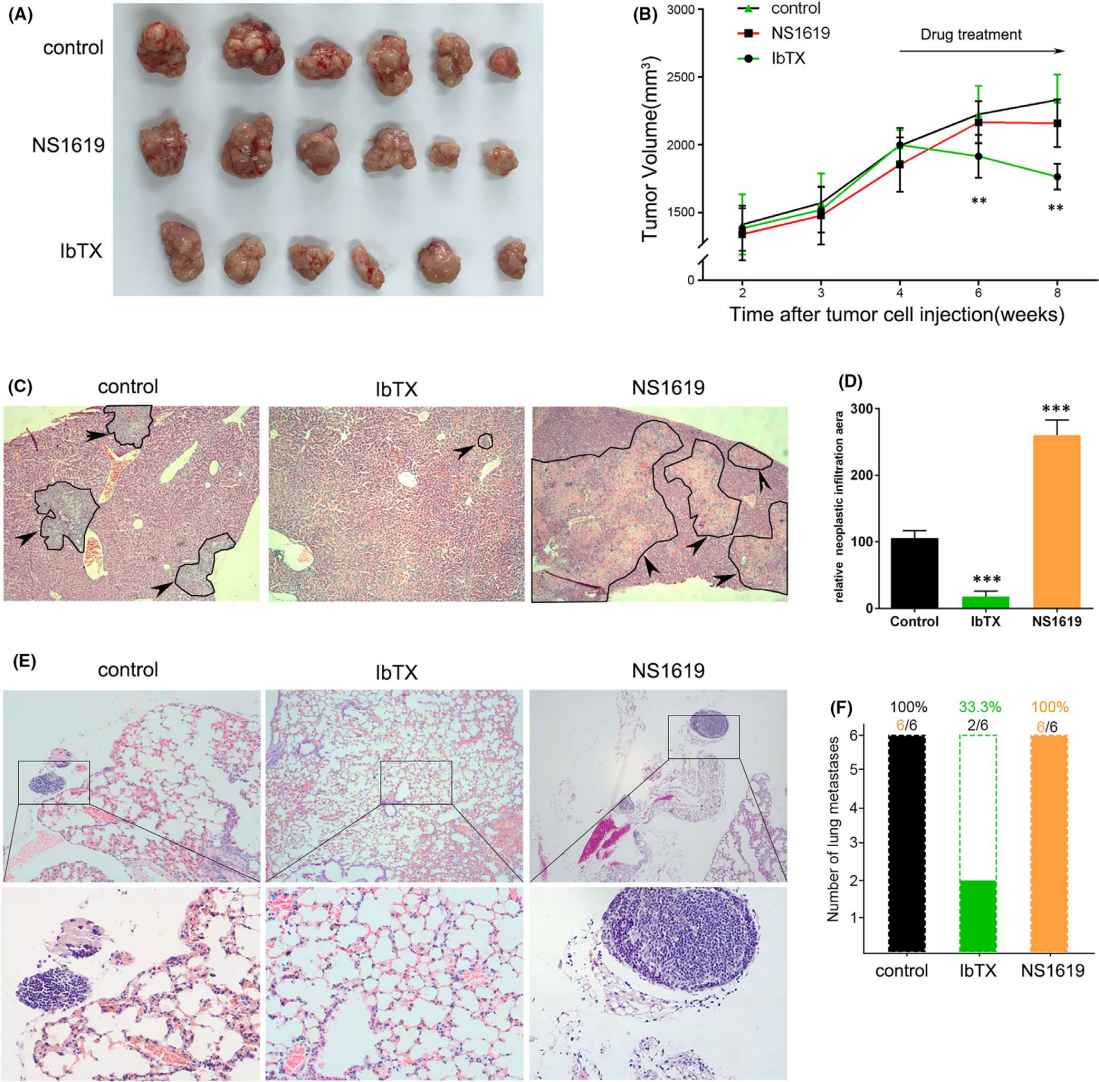
BK channel blocker and opener regulate the growth of HCC cell xenografts in mice. (A) Images of xenografts in the control (top), NS1619 (middle) and IbTX groups by the end of the experiment. SMMC‐7721 cells were injected subcutaneously into nude mice (n = 6 for each group), and the tumours were isolated 8 weeks later. (B) Tumour growth curve of SMMC‐7721 cells in nude mice before and after the treatment of the drugs (n = 6, *p* < 0.01). (C) NS1619 treatment promotes while IbTX restrains HCC cell colonization in mice liver, representative HE staining images of mouse liver tissues in control, NS1619 and IbTX groups from left to right. Neoplastic infiltration area indicated by the black arrow. (D) Quantitative analysis of relative neoplastic infiltration area for the control, NS1619 and IbTX groups. (E) Blocking BK channels in HCC cells inhibits while opening BK channels promotes its lung metastasis, the HE image of mouse lung tissues in the control, IbTX and NS1619 groups. Mouse lung tissues with HCC metastasis were enlarged in the lower panels. (F) Quantitative analysis of lung metastasis with HCC in the three groups

In addition to examine the biological functions of BK channel *in vivo*, we also assessed the effects of BK channel modulators using a xenograft transplantation model in nude mice. We subcutaneously transplanted the same number of SMMC‐7721 cells into nude mice respectively. Four weeks later, the mice were treated with PBS, NS1619 and IbTX. We monitored the tumour growth over 8 weeks before sacrificing the mice. At the end of the experiment, the tumours in IbTX group were much smaller than those in the control and NS1619 groups (Figure 6A). We also observed that the tumours in IbTX group began to shrink, while those in the control and NS1619 groups steady increased in size after the drug treatment (Figure 6B). The difference in tumour size between the control and IbTX groups can be easily observed at 6th and 8th week time point on the tumour growth curve (*p* < 0.01, Figure 6B). All six mice in the control, IbTX and NS1619 groups developed HCC liver metastasis (Figure 6C). Moreover, the neoplastic infiltration area of NS1619 group was twice that of the control group. In contrast, the neoplastic infiltration area of IbTX group was significantly smaller than that of the control group. (Figure 6D). Representative haematoxylin‐ and eosin (HE)‐stained images of lung tissues from different experimental groups are shown in Figure 6E, with tumour cells aggregation observed in the lower panel of the control and NS1619 groups. All the mice in the control and NS1619 groups developed lung metastases of HCC. However, only two of six mice in the IbTX group were found with little lung metastases (Figure 6F).

## 4 DISCUSSION

HCC has long been a serious health problem worldwide, especially in Asia.^1^ Great efforts have been made to identify effective targets for HCC diagnosis and therapy. In this context, progress has been achieved in cancer driver genes, related signalling pathways, cancer immunotherapy checkpoints that underlie the possible mechanisms for carcinogenesis. However, in most cases, HCC is not fully cured. It has been well established that disruption of ion channel function can affect membrane potential control, cell volume regulation, cell proliferation, cell death and cell migration.^26^ Moreover, an increasing number of studies have shown the involvement of ion channels in the development and progression of various cancers.^8^, ^9^

The results of this study showed that the BK channels were functionally expressed in HCC cells by using qPCR and electrophysiology recordings as another example of a BK channel located on non‐excitable tissues.^27^, ^28^ This finding was also consistent with that by Zhou et al. who also observed BK channels expression in HCC cells.^29^ Before our research on BK channels in HCC, previous studies reported that BK channels in various cancer cells such as triple‐negative breast cancer cells,^18^ neuroblastoma cells,^19^ human glioblastoma cells^21^, ^22^ and human astrocytoma cells.^30^ We examined BK channel expression in HCC patients and assessed the association of its expression with overall survival, with data obtained from online TCGA library. BK channel showed a higher expression in tumour tissues (T) compared to their non‐tumorous counterparts (NT, Figure S5.a). Besides, patients with high KCNMA1 expression showed a significantly worse prognosis than those with low KCNMA1 expression (Figure S5b).

Furthermore, we observed decreased HCC cell proliferation following the treatment of BK channel blockers, TEA and IbTX. Previous studies demonstrated that blockage of BK channels inhibited HCC^29^ and human astrocytoma^30^ cell proliferation. However, some reports indicated that blocking BK channels did not affect cancer cell proliferation^31^ or that opening BK channels selectively induced cell death in triple‐negative breast cancer.^18^ Together, these findings suggest that BK channels might have different effects in different cancers. The mechanisms underlying the effect of BK channels on cancer cell proliferation require further investigation. Normal cell cycle progression is crucial for the living of multicellular organisms and is closely associated with cell proliferation, stem cell renewal and cell death.^32^ Bioelectrical modulation of cell cycle progression is an indispensable way in which ion channels participate in cell cycle control. BK channel modulators altered cell cycle distribution in SH‐SY5Y neuroblastoma cells with different patterns.^19^ Similarly, our results showed that closing or opening BK channel by different compounds accordingly increased or reduced proportion of cells in G2 phase. Interestingly, Nadezhdin et al. observed the cell cycle‐dependent expression of BK channels in human mesenchymal endometrial stem cells.^33^ Therefore, BK channels are involved in cell cycle progression in cancer cells.

One of the challenges of HCC is its high metastasis ability, which causes great patient suffering and poor prognosis. Therefore, it is important to find effective methods to prevent HCC metastasis. In this study, we found that blockage of BK channels reduced cancer cell migration and invasion both *in vitro* and *in vivo*. Our findings support previous observations that BK channel activation contributed to PL‐induced mesenchymal stem cell migration,^20^ while BK channel blockage inhibited hypoxia‐induced migration in glioblastoma cells.^21^, ^22^ Based on these evidences, it is *bona fide* that BK channels play a crucial role in regulating cancer cell migration. We propose two possible explanations for the role of BK channels in the migration control of HCC cells. First, BK channels modulate cancer cell migration through reduced cell‐cell contact by regulating the expression of EMT related proteins such as E‐cadherin, Vimentin and N‐cadherin. However, gene knockdown or knock out studies are required to confirm the involvement of BK channels in EMT process, which can lead to metastable cellular phenotypes combining both epithelial and mesenchymal characteristics.^4^, ^23^, ^34^, ^35^ Second, ion channels facilitate neoplastic cell migration by modulating cell volume, which is necessary when cells migrate through narrow spaces of basal membrane.^36^ Cancer cells express several types of K^+^ channels including BK channels, which contributes to their migration.^37^, ^38^ Because of their large conductance, opening of BK channels leads to coordinated K^+^ and Cl^−^ efflux, causing osmotic water release from the cytoplasm to decrease the volume of the migrating cell. Direct experiment evidence of cell volume change by blocking or opening BK channels is needed in the future studies. Nevertheless, the present study reported encouraging results regarding the role of BK channels in the migration control of HCC cells and described a novel, pharmacological accessible site for the treatment of HCC.

## 5 CONCLUSIONS

In conclusion, our results showed that BK channels are functionally expressed in HCC cells and are related to the neoplastic phenotype of HCC. We discussed the comprehensive role of BK channels in HCC, which might provide direction for future studies on the implication of BK channels blockers in other cancers. BK channels may be a potential drug candidate for pharmacotherapy for HCC.

## CONFLICT OF INTEREST

All authors confirm that there are no conflicts of interest.

## AUTHOR CONTRIBUTIONS

**Yuan He:** Data curation (lead); Funding acquisition (supporting); Investigation (lead); Methodology (equal); Resources (equal); Visualization (equal). **Yingying Lin:** Formal analysis (lead); Investigation (lead); Software (equal); Validation (lead). **Fei He:** Funding acquisition (supporting); Investigation (supporting); Methodology (supporting); Resources (equal); Validation (equal). **Lijuan Shao:** Data curation (supporting); Formal analysis (equal); Investigation (equal); Validation (equal); Visualization (supporting). **Wei Ma:** Data curation (equal); Investigation (supporting); Visualization (supporting). **Fei He** ^5^: Data curation (lead); Funding acquisition (lead); Investigation (lead); Methodology (equal); Resources (equal); Visualization (lead).

## ETHICS APPROVAL AND CONSENT TO PARTICIPATE

All animal experiments were approved by the Animal Care and Use Committee of Shenzhen People’s Hospital and conducted in accordance with their principles of animal welfare.

## CONSENT FOR PUBLICATION

All authors read and approved the final manuscript.

## Supporting information

Fig S1

Fig S2

Fig S3

Fig S4

Fig S5

Table S1

Supplementary Material

## Data Availability

The data sets used and/or analysed during the current study are available from the corresponding author on reasonable request.
