# Supplementary figures and images for: Role for calcium‐activated potassium channels (BK) in migration control of human hepatocellular carcinoma cells
Source: J Cell Mol Med. 2021 Sep 12;25(20):9685–96. doi: 10.1111/jcmm.16918 (PMC8505838; doi:10.1111/jcmm.16918)

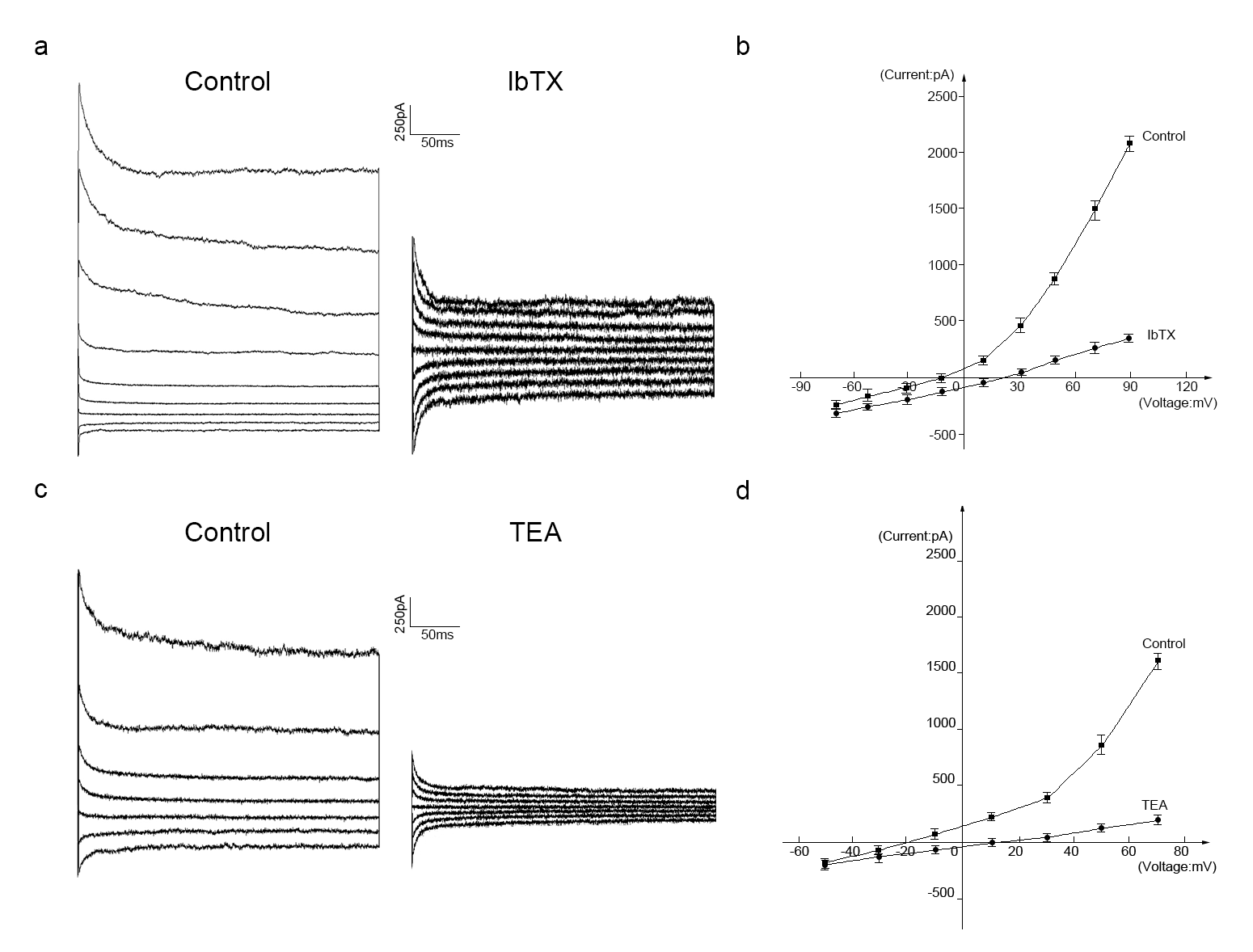

Supplement: Supplementary file 1 — Fig S1 [file JCMM-25-9685-s003.tif]

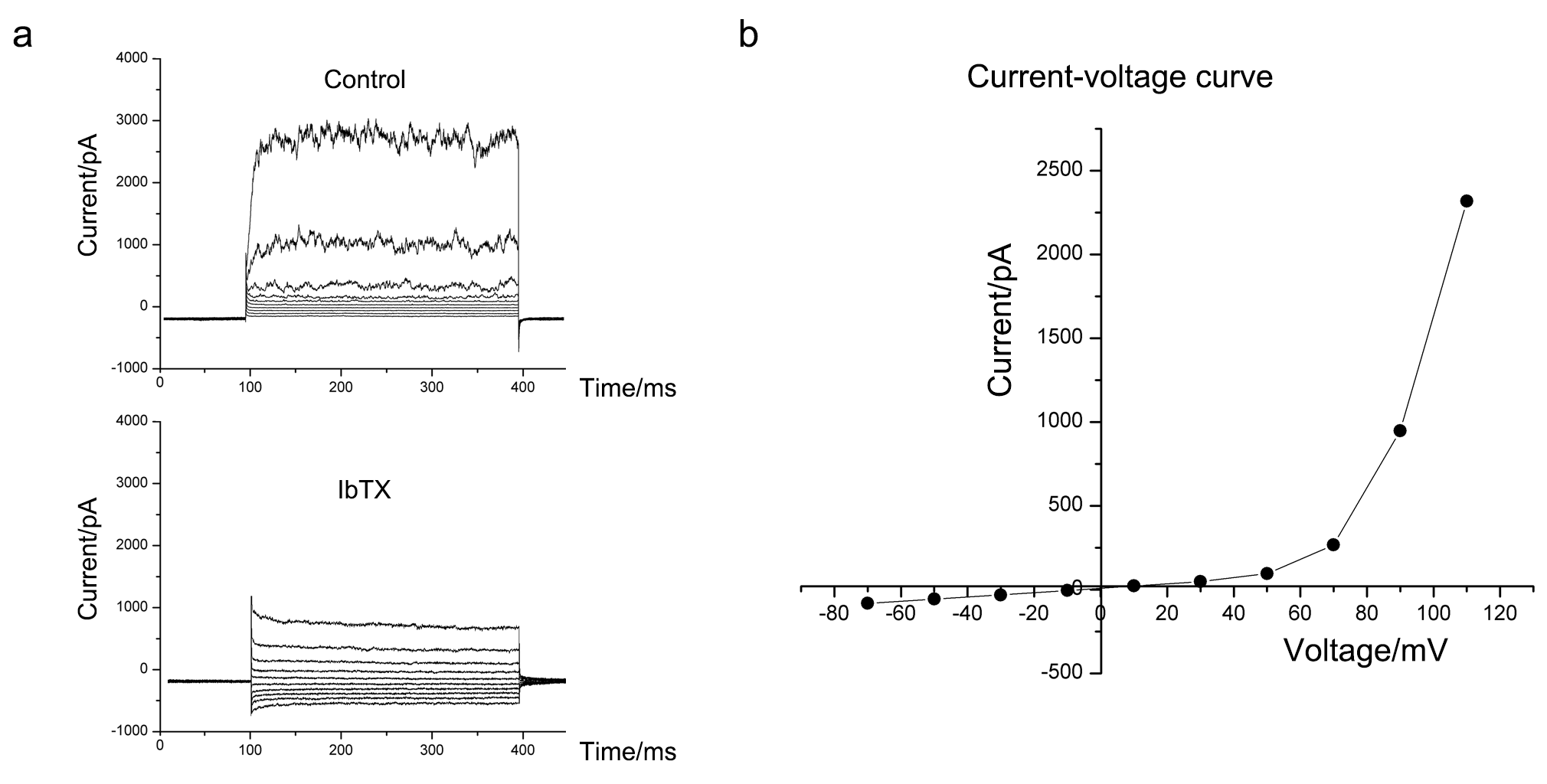

Supplement: Supplementary file 2 — Fig S2 [file JCMM-25-9685-s007.tif]

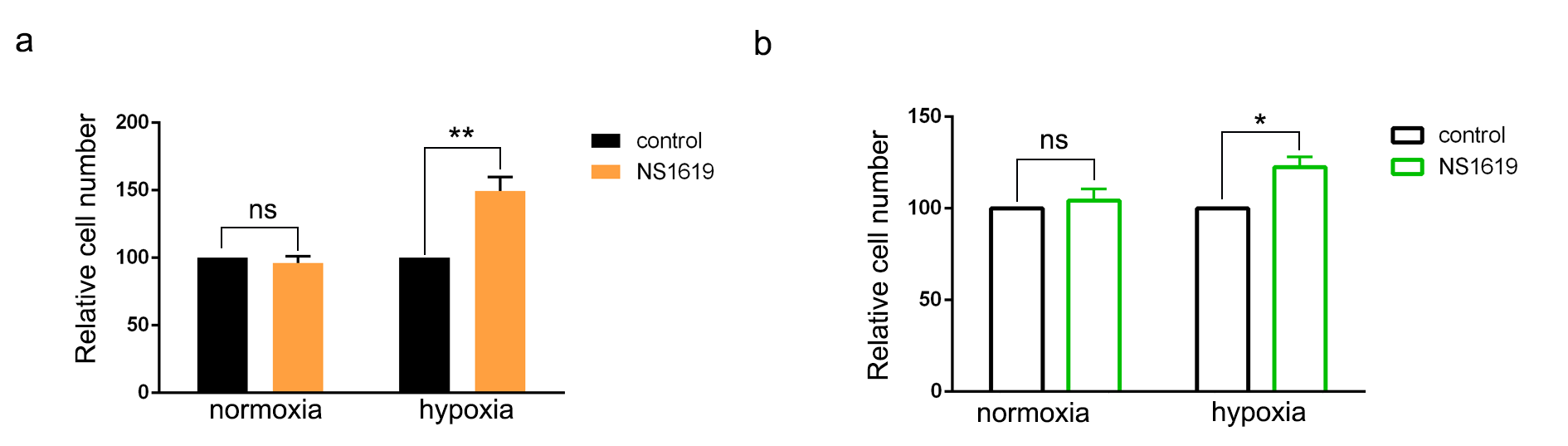

Supplement: Supplementary file 3 — Fig S3 [file JCMM-25-9685-s005.tif]

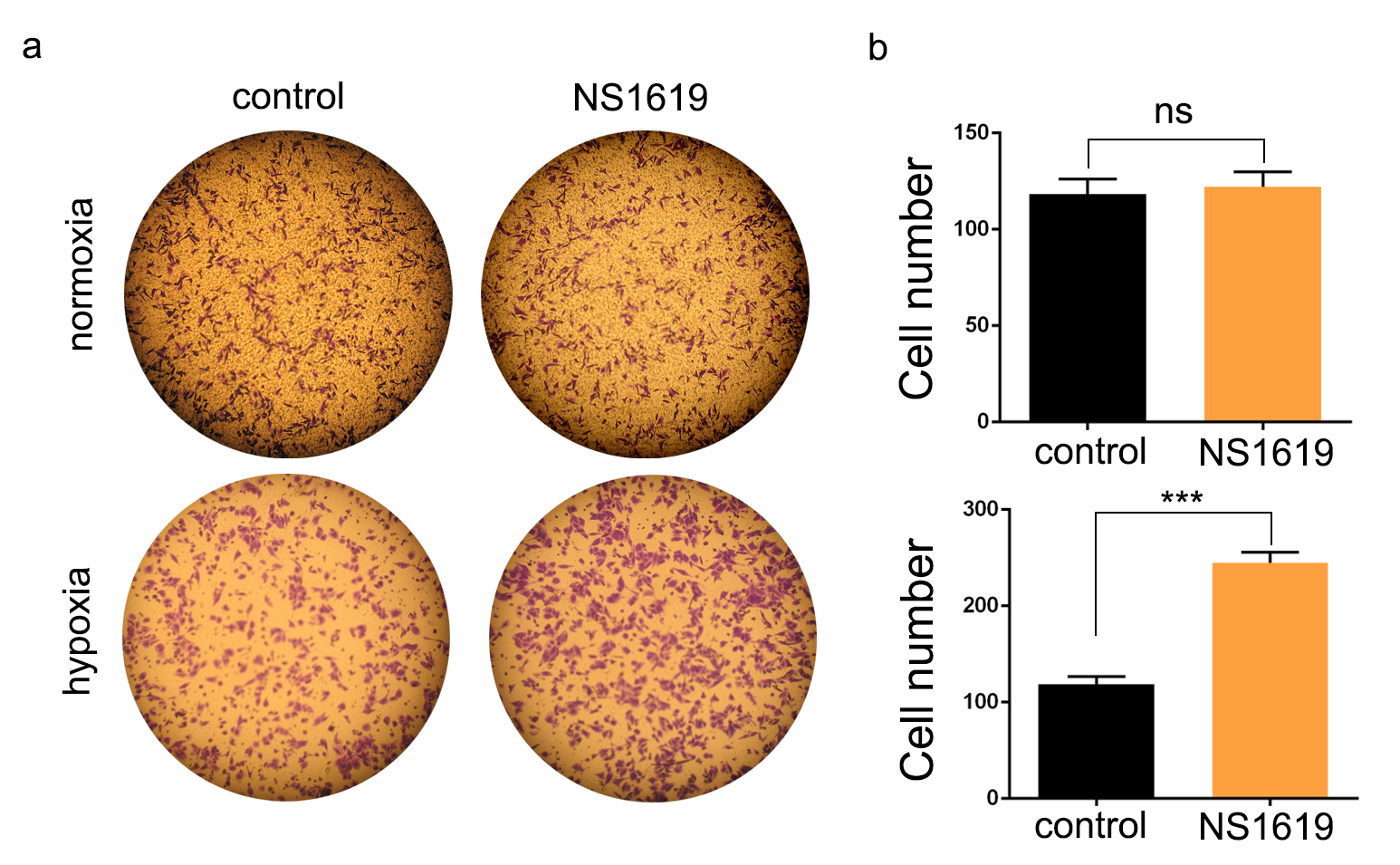

Supplement: Supplementary file 4 — Fig S4 [file JCMM-25-9685-s001.tif]

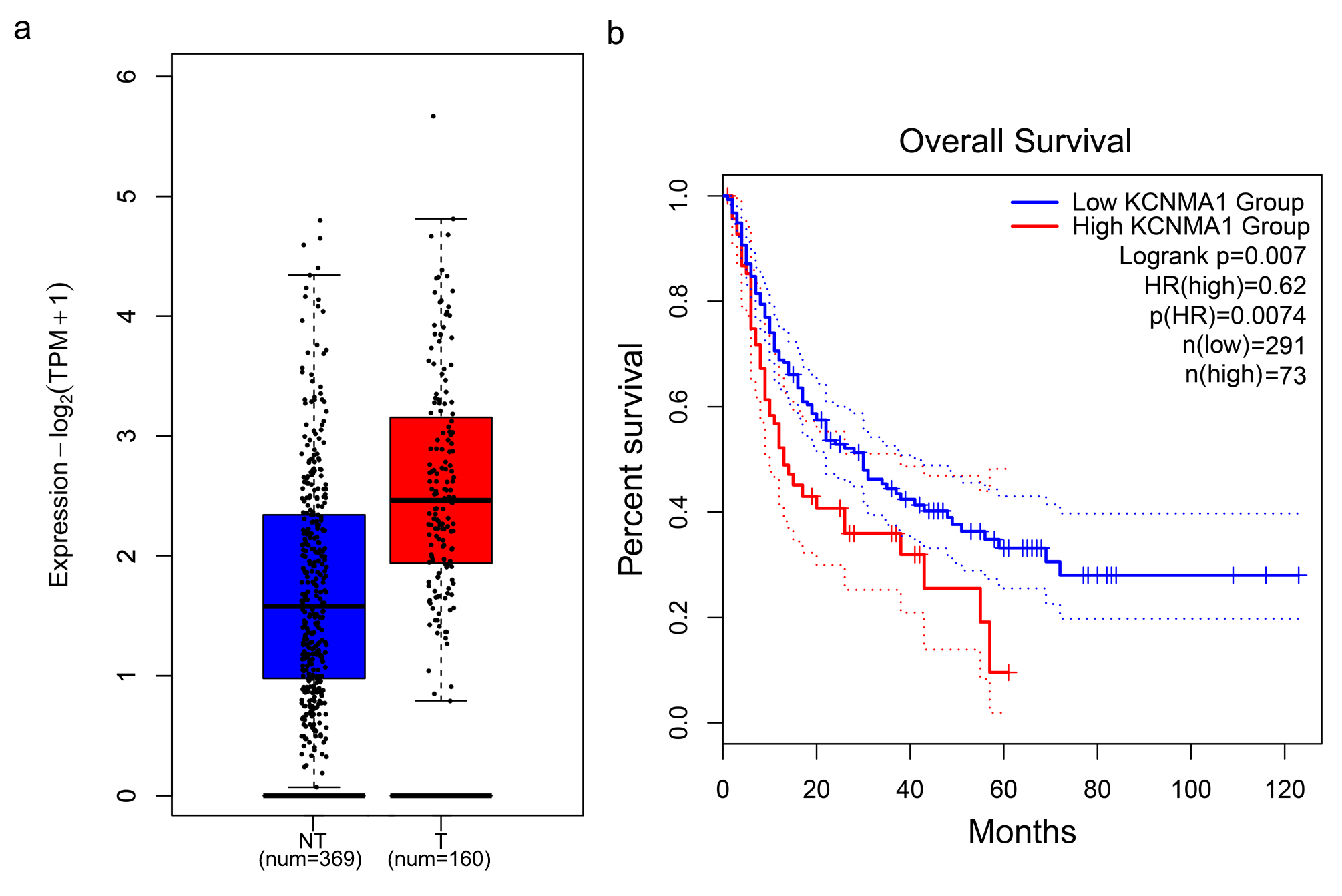

Supplement: Supplementary file 5 — Fig S5 [file JCMM-25-9685-s002.tif]
