# Supplementary material for: Role for calcium‐activated potassium channels (BK) in migration control of human hepatocellular carcinoma cells
Source: J Cell Mol Med. 2021 Sep 12;25(20):9685–96. doi: 10.1111/jcmm.16918 (PMC8505838; doi:10.1111/jcmm.16918)
Supplement: Supplementary file 6 — Table S1 [file JCMM-25-9685-s006.docx]

Supplement Table 1: Primers for the qPCR quantification of K^+^ channels in HCC cell lines.

| Gene | Primer sequence | |
| --- | --- | --- |
| ACTB | Forward | CATGTACGTTGCTATCCAGGC |
|  | Reverse | CTCCTTAATGTCACGCACGAT |
| KCNQ1 | Forward | ATCTGCGTAGCTGCCAAAC |
|  | Reverse | GCGTAGCTGCCAAACTCGAT |
| KCNQ2 | Forward | GCTCTGATGCTGACTTTGAGGC |
|  | Reverse | TGCTGACTTTGAGGCCAGG |
| KCNQ3 | Forward | ATGGTGGCCAGTGTGATCAG |
|  | Reverse | GGGCATCAGCATAGGTCTCAA |
| KCNQ4 | Forward | TTGATTCGTCCCAGCATGTCCA |
|  | Reverse | TTCGTCCCAGCATGTCCA |
| KCNE1 | Forward | TGGTACTGGGATTCTTCGGC |
|  | Reverse | AGGAAGGTGTGTGTTGGGTTG |
| KCNE2 | Forward | GCTGAGGCTTGTGTGCAACC |
|  | Reverse | GGATGGTGGCCTTCGATTC |
| KCNE3 | Forward | ACCAATGGAACGGAGACCTG |
|  | Reverse | ACTACGCTTGTCCACTTTGCG |
| KCNMA1 | Forward | CATTTGGTGGAGAATTCAGG |
|  | Reverse | GATGAAGAAGACCATGAAGAG |
