# Supplementary material for: Role for calcium‐activated potassium channels (BK) in migration control of human hepatocellular carcinoma cells
Source: J Cell Mol Med. 2021 Sep 12;25(20):9685–96. doi: 10.1111/jcmm.16918 (PMC8505838; doi:10.1111/jcmm.16918)
Supplement: Supplementary file 7 — Supplementary Material [file JCMM-25-9685-s004.docx]

Figure S1. Whole-cell BK currents were recorded in Huh7 cells. (a) Representative whole-cell currents elicited by step pulses from –70mV to +90 mV for 300 milliseconds with 20mV increment. (b) Whole-cell current-voltage relationships in Huh7 cells in the absence and presence of 10nM IbTX. (c) Representative whole-cell currents evoked after voltage steps from –70mV to +70 mV for 300 milliseconds with 20mV increment. (d) Whole-cell K^+^ currents were potently blocked by the specific inhibitor of BK channel, TEA (10mM).

Figure S2. Whole-cell BK currents were recorded in LO2 cells. (a) Representative whole-cell currents evoked by step pulses from –70mV to +110 mV with 20mV increment, for control group (upper panel) and IBTX group (lower panel). (b) Current-voltage curve for the whole-cell current in the control group.

Figure S3. Effect of BK channel opener NS1619 on the cell proliferation of HCC cells. (a)Quantitative analysis of SMMC-7721 cell proliferation in the control and NS1619 group under normoxic and hypoxic conditions. (b)Quantitative analysis of LO2 cell proliferation in the control and NS1619 group under normoxic and hypoxic conditions. Cell proliferation assay was done using CCK-8 kit. ns, no significance, p > 0.05; *, p < 0.05; **, P < 0.01; ***, p < 0.001.

Figure S4. BK channel opener NS1619 promotes HCC cell migration under hypoxic condition. (a) Representative SMMC-7721 cell images of transwell assay in control and NS1619 groups under normoxic and hypoxic conditions. (b) Statistical description of cell migration ability of SMMC-7721 cells in control and NS1619 groups. Each experiment was done in triple replicate, and p<0.001 was marked ***.

­Figure S5. BK channel expression in liver cancer tissues and non-tumorous tissues from HCC patients. (a) BK channel expression was increased in tumour tissues (T, n=160) compared to adjacent non-tumorous counterparts (NT, n=369). (b)Kaplan-Meier survival curves of HCC patients after hepatectomy. Patients with high KCNMA1 expression had a significantly worse prognosis than those with low KCNMA1 expression (log-rank test, p = 0.007).
